# Cognitive Functioning, Life Satisfaction, and Their Relationship with the Financial Attitudes of Older Individuals Who Participate in an Active Aging Program

**DOI:** 10.3390/bs10120189

**Published:** 2020-12-10

**Authors:** Claudia Idárraga-Cabrera, Jorge-Manuel Dueñas, Marina-Begoña Martínez-González, Regina Navarro-Blanco, Marianela Denegri-Coria, Mariana Pino

**Affiliations:** 1 Department of Social Sciences, University of La Costa, Barranquilla 080002, Colombia; cidarrag1@cuc.edu.co (C.I.-C.); mmartine21@cuc.edu.co (M.-B.M.-G.); 2 Department of Psychology, Universitat Rovira i Virgili, 43007 Tarragona, Spain; 3 Center of Excellence in Economic and Consumer Psychology, University of La Frontera, Calle Francisco Salazar, Temuco 1145, Chile; reginanb@gmail.com (R.N.-B.); marianela.denegri@ufrontera.cl (M.D.-C.); 4 Faculty of Social Sciences, Autonomous University of the Caribbean, Barranquilla 080002, Colombia; mariana.pino@uautonoma.edu.co

**Keywords:** financial attitudes, active aging, life satisfaction, older people, aging

## Abstract

Life expectancy has increased in many countries throughout the world over recent years, leading to new challenges related to aging. A large part of the population is over 60 years old, and therefore studies that focus on financial autonomy and active aging are necessary. In this paper, we analyze the relationship between cognitive functioning and life satisfaction in relation to financial attitudes in a sample of 251 elderly adults (71% women) participating in an active aging program (M = 67.5, SD = 4.5). We used the Mini-Mental State Examination, the Satisfaction with Life scale, and a questionnaire about financial attitudes to gather data. Our results show that cognitive functioning and life satisfaction are related to certain financial attitudes. We also observed differences associated with gender, education level, and financial independence. According to our findings, life satisfaction should be taken into account when the financial attitudes of older adults are evaluated. The importance of support groups for the elderly is highlighted as well as of all those recreation and health programs, since they constitute a means of promoting well-being.

## 1. Introduction

Aging is a multifaceted process of maturation and decay that starts at birth and continues through to death [1]. However, some literature mainly considers it as the final stage in a person’s life cycle, which is characterized by a lower ability to adapt to one’s environment. In other words, aging entails a series of biological, social, psychological, and cultural changes that lead, for example, to dependence, cognitive rigidity, and difficulty in adapting to a changing environment [2]. 

The evolutionary characteristics of normative aging indicate that although there is a gain in knowledge, experience, patience, and perspective on life, there is also a decline in cognitive functioning skills, such as memory, attention, and information processing, which mainly becomes manifested with advanced age [3,4]. These shortcomings can be compensated for, however, by social group participation, family support, a continued lifestyle [5,6], physical exercise [7], and educational activities such as reading, all of which enable the training of cognitive skills [8,9]. Within the physical activities where balance is trained, spatial memory and orientation play an essential role in the maintenance of functional capacity [10] which is related to the quality of life of the elderly [11]; in turn, maintaining functional ability has been linked to subjective well-being in older people [12].

In addition to changes in cognitive performance, aging also implies social transformations. One example is retirement, which involves changes in structures, functions, habits, and the general organization of daily life. These changes affect a person’s sense of efficiency and competence [13]. Industrialized consumer societies therefore consider aging as something negative, emphasizing characteristics such as loss, disability, illness, exclusion, and loneliness [14]. However, as societies around the world are home to an unprecedented number of aging citizens [15], there is growing evidence of more positive attitudes toward aging as well as the developmental possibilities of this life phase. There is also a growing awareness of the challenge to respond to these needs with demographic, social, and economic policies [4,16]. 

### 1.1. Active Aging 

With the aforementioned factors in mind, the term “active” refers to the continued participation in the various overall human dimensions—for example, economic and cultural dimensions. It also refers to the ability of older retired people and those who are ill or living with disabilities to continue actively contributing to their families, partners, communities, and nations, and not just the ability to be physically active [17]. Active aging is a general concept that encompasses a semantic space in which healthy, successful, and productive aging processes are closely related. It is understood as the multidimensional process of optimizing opportunities for health, participation, and safety to improve life quality and well-being as people age. That is, it refers to a positive way of aging, or aging well [18]. Active aging enables people to fulfill their potential for physical [19], social [20], and psychological [21] well-being throughout their lives, allowing them to participate in different settings in society. It takes into account people’s needs, desires, and abilities while also providing protection, security, and adequate care when required [22]. 

Active aging programs aim to address aging challenges, both individually and in the population. These programs focus on promoting health and preventing disease, the labor market, employment, education, and social policies that support the different aging processes [23]. Active aging programs have demonstrated their benefits in promoting health [24,25,26]. More specifically, the results of a study that analyzed and compared cognitive performance, general health, positive affect, and level of cultural and social activity in a group of elderly people who participated in a university active aging program and a control group shows that elderly people who participated in the program exhibited a better performance in all the analyzed variables [27]. Similar results were reported by other authors [28], who also compared an experimental group that participated in an active aging program and a control group. The experimental group showed a significant improvement in physical activity, nutrition, cognitive performance, and life quality. Similarly, having an active life has been associated with cognitive functioning [24,29,30]. More specifically, Pereira et al. [30] identified the benefits of an active aging program with a comprehensive approach on various components of cognitive function in older adults, and the results showed that physical exercise improved motor control, spatial and visual working memory, heuristic strategic reasoning, and associated visuospatial learning. 

### 1.2. Financial Competence

Financial skills gain particular importance in adulthood and old age; indeed, people of retirement age are more likely to fall victim to fraud [31]. More specifically, the other study, with older people living in residences without dementia and with an autonomous life outside the center, reported that the more elderly and those people who scored lower on cognitive functioning, wellness, and financial planning appeared to be more vulnerable to fraud, regardless of their level of education or income [32]. Under the same line, the changes produced in cognitive and social functioning, from normal to pathological aging, may be related to deficiencies in decision-making that affect financial management [33]. It appears there may be a relationship between cognitive functioning and financial management. A longitudinal study of older people without dementia reported that those with mild cognitive impairment showed significant decreases in multiple financial skills, especially financial judgment [34].

Few empirical studies of the relationship between cognitive functioning and financial skills have focused on older people. However, some studies have reported a relationship between cognitive ability and financial literacy [35]. One’s financial capabilities appear to decline after the age of 60; however, confidence in one’s financial decision-making skills does not appear to diminish with age [36]. Several studies have also shown that those with financial literacy and greater cognitive ability tend to seek financial help from professionals [37]. It appears, therefore, that cognitive functioning is related to various financial aspects. However, these relationships have not been widely studied in older people in the context of active aging.

### 1.3. Life Satisfaction

Several studies have found links between life satisfaction and financial satisfaction [38,39]. However, as these relationships are measured by a person’s perceived financial capacity [40], the proper management of one’s finances may be related to being satisfied with life, since proper financial management promotes greater perceived purchasing power, takes precedence over risk-taking with purchases or products, and discourages people from exceeding their debt capacity [41]. One study suggested that positive financial behaviors contribute to financial satisfaction, while financial satisfaction contributes to life satisfaction [42]. Specifically, financial management behaviors influence both the quality of relationships and subjective well-being [43]. However, studies in this area have given little attention to the relationships between life satisfaction and financial attitudes in older adults participating in active aging programs. Some studies on life satisfaction, financial management, and retirement have reported that full retirement predicts greater life satisfaction only for those with adequate financial management [44]. However, it appears that when financial management enables money to be spent on leisure rather than just on housing and basic needs, full retirement is indeed related to satisfaction with life [36].

### 1.4. The Current Study

Our main objective was to analyze the relationships between cognitive functioning and life satisfaction vis-à-vis financial attitudes in older adults who have participated in an active aging program for over five years. To do so, we also took into account the above literature.

In the literature, we expected to observe a relationship between cognitive functioning and different financial attitudes [33,35], since financial management requires a series of cognitive aspects that may decrease during aging. However, this has been studied very little in older individuals participating in active aging programs. Based on approximations in previous studies, we also expected to find a relationship between life satisfaction and different financial attitudes [32,38], since suitable financial attitudes can generate psychological benefits, such as fewer financial worries. Finally, we also expected to find that most participants had adequate cognitive functioning, since several studies show that cognitive deterioration is slower in people who are actively aging [27,28,45].

Taking into consideration that there are few studies on the subject, this study is relevant because it allows us to know the relationships between cognitive functioning, different financial attitudes, and satisfaction with life in older people. This knowledge is essential to being able to implement programs to promote active aging and financial autonomy in people aging in developing countries. Given this, South American countries’ life expectancies have been increasing in recent years [46].

## 2. Materials and Methods

### 2.1. Participants

We used intentional non-probability sampling in which we selected 251 adults (71% women) between the ages of 58 and 75 (M = 67.5, SD = 4.5) in Barranquilla (Colombia), 93.3% of whom asserted that they did not have a credit card. All participants were considered to be active in Life Centers. These are social integration centers run by town halls through Colombia’s Ministry of Health and Social Protection. Life Centers ensure inclusion and comprehensive care for the elderly. In these Life Centers, care consists of regular medical evaluations which include physical, social, and cognitive assessments as well as leisure activities. The participants attend these social integration centers where they take part in activities to develop their intellectual and social skills. We established the sample size following the provisions of Daniel and Cross [47] for finite populations, (population size = 8000, the margin of error = 10%, confidence level = 95%) = sample adequate (95), the size of the population corresponds to the number of older adults who were part of the Life Center in the city of Barranquilla at the time the sample was collected. To select the sample, we established the inclusion and exclusion criteria. People with the following characteristics were invited to participate: being over 58 years old, being a user of the active aging programs at the Life Centers, and having given informed consent. For their part, people with psychiatric or neurological diagnoses were not invited to participate. Table 1 shows the characteristics of the sample.

### 2.2. Procedure

The study was approved by the ethics committee of the Vice Chancellor for Research and Knowledge from the Autonomous University of the Caribbean (Colombia) and conducted following the Declaration of Helsinki. The data were collected anonymously and data custody was ensured. Participants were invited to participate via an informative letter. People who showed interest in participating re-read the informed-consent form and were invited for an interview to ask as many questions as they liked. After the interview, they had one week to decide on whether they wished to participate in the study or not. This was to make sure that people did not feel pressured to participate.

When people agreed to participate in the study, they had to sign the informed consent form, and it was explained again to them that they could withdraw whenever they wanted. Once the informed consent form was signed, the participants were summoned to the same day center where they completed their activities. The tests were applied individually by a professional psychologist who is an expert in gerontology and familiar with this type of questionnaire.

### 2.3. Measures

To evaluate cognitive functioning, we used the Mini-Mental State Examination (MMSE) [48], according to the authors; the finest cut-point for the cognitive deficits associated with dementia was 24 which gives good sensitivity (90%) and specificity (75%), with an area under the Receiver Operating Characteristic (ROC) curve of 0.92. The MMSE is the most common screening test for evaluating the mental state of elderly people. MMSE investigates the following cognitive facets: Orientation, Registration, Attention and Calculation, Recall, and Language, and has adequate psychometric properties. We used the Spanish version of this instrument [49].

We also used the Spanish version of the Satisfaction with Life Scale (SWLS) [50], which was developed by Atienza, Pons, Balaguer, and García-Merita [51]. This version has suitable psychometric properties and a reliability of 0.84, indicating good internal consistency. The questionnaire is unifactorial and consists of five Likert-scale items with five alternative answers ranging from (1) totally disagree to (5) totally agree.

To analyze financial attitudes, we used an ad hoc questionnaire based on the one developed by Carpenter and Moore [52]. This questionnaire consists of seven questions on basic personal financial attitudes, with five options for responses based on a 5-point Likert scale ranging from (1) strongly disagree to (5) strongly agree. The questions are shown in Table 2.

We also used a sociodemographic questionnaire. On the first page the participants were asked about gender, age, education level, employment status, income, and credit card ownership. There was also one question with a dichotomous answer.

### 2.4. Statistical Analysis

Statistical analyses were conducted using IBM SPSS Statistics Version 26.0 at a significance level of 0.05. The normality of the data was analyzed using the Kolmogorov–Smirnov test. Statistical analyses were conducted in the following order. First, the descriptive procedures (mean and standard deviation) were performed. Second, a *t*-test examined gender differences in all variables and differences based on the question with a dichotomous response “Do you describe yourself as financially independent?” Cohen’s *d* was used to determine the effect size as follows criteria: 0.2 < *d* < 0.5 = small; 0.5 < *d* < 0.8 = medium; *d* > 0.8 = large [53]. Third, Analysis of Variance (ANOVA) was used to determine significant differences according to the degree of performance in the MMSE, we use the following cut-points: more than 24 points = Normal; between 19 and 23 points = Mild impairment; between 10 to 18 points = Moderate; Equal to or less than 9 points = Severe. We also used ANOVA to analyze the differences in means according to education level in all study variables, Tukey’s honest significant test was used to determine multiple comparisons. Ultimately, the degree of association with the MMSE and life satisfaction with the financial attitude questions was also analyzed using Pearson’s correlation coefficient.

## 3. Results

Table 2 shows the descriptive statistics for the measures across the whole sample—women and men. These data reflect significant differences in the MMSE, with significantly higher means in men (Mean = 29.49; Standard Deviation = 2.82) than in women (Mean = 28.85; Standard Deviation = 3.56) and a small effect size (*t*-test (_223_) = 2.45 *p* < 0.01; effect size: Cohen’s *d* = −0.19) for the remaining variables. These differences were not significant.

We analyzed the performance of our sample on the MMSE and found three performance groups: (1) mild impairment (10.7%); (2) moderate (41.3%); (3) normal (48%); we did not obtain any participant with severe results. To analyze possible differences based on performance in the MMSE, an ANOVA was carried out, and the results do not show significant differences.

When we conducted an ANOVA to analyze the differences in means according to the educational level in all study variables, we found significant differences in the MMSE (F_(223)_ = 3.74; *p* ≤ 0.01). Pairwise mean comparisons using Tukey’s HSD test showed significant differences between the group with no study (M = 27.00; SD = 3.21) and the group with vocational study (M = 29.83; SD = 3.15), with a large effect size (difference = 2.83; *p* < 0.01; Cohen’s *d =* 0.87). Differences were also found between the group with no study and the group with university study (M = 30.68; SD = 3.26), with a large effect size (difference = 2.83; *p* < 0.01; Cohen’s *d =* 1.14). No significant differences were found for the other variables. We also conducted a *t*-test based on the question “Do you describe yourself as financially independent?”, which had a dichotomous (yes/no) response. The results show significant differences in the responses to question 1, with a higher average in people who believe they are ready to face their financial future (Yes: M = 3.71, SD = 0.84.; No: M = 3.34; SD = 1.16; *t_(168)_* = 2.23; *p* < 0.01.; Cohen’s *d =* 0.38). Significant differences were also found in the responses to question 3 (Yes = 3.15, SD = 1.20., No = 3.69, SD = 1.00; *t_(169)_* = −3.11; *p* < 0.01.; Cohen’s *d* = −0.48) and question 7 (Yes = 3.26, SD = 1.20.; No = 3.71, SD = 1.04; *t_(169)_* = −2.49; *p* = 0.001; Cohen’s *d* = −0.39), with higher averages for people who consider themselves unprepared for their financial future.

Table 3 shows the Pearson correlations between the MMSE and life satisfaction in relation to the questions on financial competencies. As we can see, the MMSE correlates positively and significantly with questions 5 and 7. However, life satisfaction correlates positively and significantly with questions 1, 2, 5, and 7. According to Guilford [54], these correlations are moderate.

## 4. Discussion

The main aim of this study was to analyze the relationships between cognitive functioning and life satisfaction vis-à-vis financial attitudes in older adults participating in an active aging program. Our data revealed gender differences in the MMSE, with higher means for men. These data are consistent with previous studies on older people without severe cognitive impairment [55,56]. However, women tend to perform less well than men in cognitive functioning at the onset of aging but then stabilize [55]. The average age of our sample was 67.5, with a standard deviation of 4.5, which is in accordance with our results. More specifically, according to Navarro-González and Calero [57], when educational variables are tested, men perform better than women. These results illustrate the importance for the neurocognitive results of checking sociodemographic variables during the period of advanced adulthood.

When we classified the participants’ performance on the MMSE, we obtained only three categories: mild cognitive impairment, slight cognitive deficit, and cognitive normality. Our data showed that 89.3% of the sample had a slight deficit of cognitive normality. These data are consistent with previous studies that reported good cognitive functioning in those participating in active aging programs [21,24,58]. Our data also support previous studies on the potential benefits of active and prolonged aging. In fact, our study has shown that the neurocognitive processes of socially active older adults are not greatly deteriorated. According to Durán [59], social interaction can be a protective health factor since it involves connecting with others and a constant level of social and cognitive activities.

Our results indicate that cognitive status was associated with education level, and specifically with higher education and higher performance. The MMSE has been widely used in the older adult population and previous studies have found that it is sensitive to demographic conditions, such as culture and education [60]. These variables are known to strongly influence performance on neuropsychological tests and the brain structure of older adults from normal functioning to dementia [61,62]. Cognitive stimulation, academic and educational factors, social activities, and work activities favor the development of a cognitive reserve and are considered significantly protective factors against the manifestations of deterioration that may appear in adulthood [63]. High cognitive training from experience therefore enables these people to apply flexible neurocognitive strategies and find alternative adaptive solutions to everyday problems [64].

When our participants were asked about their financial independence, we found that those with a self-perception of financial independence felt more prepared to meet their financial challenges. On the other hand, those whose self-perception of financial independence was low scored higher in seeking constant help to meet their financial challenges. Although we do not know of any other studies that have analyzed these aspects, similar studies have similar findings [65,66]. Financial independence seems to provide older people with the security they need to make sound personal financial decisions and to detect fraud or deception [32]. However, although seeking help when making financial decisions could protect against fraud, when this help is constant and is provided on basic economic issues, it may affect the person’s subjective well-being. More studies are needed to clarify these issues.

Our results showed positive correlations between the MMSE results and the ability to rationally organize one’s budget for monthly expenses. These findings were as expected since organizing money requires basic cognitive skills. They also agree with those of previous studies with different age groups. A longitudinal study, for example, found a relationship between financial education and improved cognitive functioning [67]. The MMSE also obtained a positive relationship with the ability to pay basic expenses. This was also as expected since the timely payment of bills requires the cognitive abilities of recall and planning. This information could be helpful when active aging programs are organized or health and social care are provided for the elderly.

Finally, we found significant and positive relationships between life satisfaction and four of the seven financial skills we studied. These data fall in line with those of similar studies. Specifically, suitable financial attitudes are related to financial satisfaction [68], which is in turn related to satisfaction with life [38,39]. Similarly, according to Xiao, Tang, and Shim [42], positive financial attitudes help people to perceive greater satisfaction with their finances and therefore they have better subjective well-being [43].

## 5. Conclusions

The operational process of an individual is linked to social activities, but there is a high probability that as age increases, participation in these types of groups and networks that provide emotional support will decrease [69]. For this reason, support groups for old age are essential, as well as recreation and health programs. These groups are a means of prevention and also contribute to improving emotional, physical, family, and social well-being. Of particular interest, this study highlights the need to generate conditions of equality for women, who, given the historical-social circumstances related to home life, usually have fewer opportunities for education, job development, and social interaction. This places them in a position of risk of further cognitive decline in old age. This study’s population corresponds to a generation that experienced a high level of gender inequality. Although the participants showed differences in scores that are within normality, these scores show that women are at a disadvantage in the aging process.

However, the present study’s results suggest that there is a relationship between satisfaction with life and some financial attitudes in older adults belonging to active aging programs. Therefore, subjective well-being variables, such as satisfaction with life, must be considered when attitudes and financial capacities in the last years of life are evaluated. Our data suggest that people with a perception of financial self-sufficiency feel prepared to face future challenges that would affect their life projects in old age. Our data also suggest that people who participate in active aging programs tend to have adequate levels of satisfaction with life as well as cognitive functioning. These data can have an impact on financial attitudes. In turn, financial attitudes can be a protective factor against fraud or financial mismanagement.

### 5.1. Practical Implications

Belonging to a group that promotes training cognitive abilities in the evolutionary stage of older adulthood is a protective factor against aging conditions, in which there is evidence of a decline in some neurocognitive processes. This is because recreational, pedagogical, creative, and social stimulation activities are carried out in these active aging groups for the elderly. These activities stimulate brain structures for executive functioning training, which is essential for adapting to the external environment. In addition to the processes mentioned above, older adult groups favor autonomy and the ability to resolve daily situations independently. This is an advantage in family and social structures, since the older adult can act without care or surveillance from another individual.

All of these contribute to the cognitive reserve, which influences decision-making and impacts the financial dimension because elderly people can keep their perspective of the relationship between income and expenses for maintaining a social nucleus. The results of this study can be taken into account in programs for promoting emotional health and preventing fraud and deception.

### 5.2. Study Limitations

Our study has certain limitations, one of which concerns the use of an ad hoc questionnaire to assess financial attitudes. This was due to the lack of questionnaires adapted for Spanish speakers. To enable a data comparison and further progress in the subject area, future studies should focus on designing standardized questionnaires that are adapted to Spanish speakers. In addition, our methodological design focused on older adults who belong to an active aging program, and therefore did not include older adults who do not participate in these programs, which would have allowed us to analyze differences in relation to this variable. Finally, our sample is primarily made up of women, although our data go in the direction of previous studies. Most studies with older adults have limitations, so studies are necessary to allow the analysis of large samples of older adult men.

## Figures and Tables

**Table 1:** Characteristics of the sample.

Educational Level	N	%	Occupation	N	%	Socioeconomic Level	N	%	Cognitive Status	N	%
None	20	8.0	Retired	157	62.5	Low-low	122	48.6	Mild impairment	24	10.3
Primary school	138	55.0	Employed	17	6.8	Low	77	30.7	Moderate	93	41.3
High school	17	6.8	Independent	40	15.9	Low-middle	46	18.3	Normal	108	48
Vocational education	50	19.9	Home Care	34	13.5	Middle	6	2.4			
College/ University	26	10.4	Prefer not to answer	3	1.2						

**Table 2:** Descriptive statistics.

Variable	Overall Sample	Women	Men
	Mean (SD)	Mean (SD)	Mean (SD)
MMSE	29.18 (3.38)	28.85 (3.56)	29.49 (2.82)
Satisfaction with life	17.67 (2.99)	17.85 (2.96)	17.27 (3.03)
Question 1: I am ready to face my financial future.	3.58 (1.00)	3.56 (1.05)	3.64 (0.90)
Question 2: I have enough financial security to face unforeseen expenses immediately.	2.81 (1.18)	2.71 (1.19)	3.04 (1.15)
Question 3: I need outside help to manage my basic finances.	3.43 (1.15)	3.38 (1.18)	3.53 (1.08)
Question 4: I attend financial planning seminars/courses.	2.62 (1.23)	2.69 (1.26)	2.47 (1.15)
Question 5: I am able to rationally organize my budget for monthly expenses.	3.82 (0.87)	3.82 (0.90)	3.84 (0.80)
Question 6: I am able to pay my basic consumption.	4.04 (0.76)	4.06 (0.77)	3.99 (0.47)
Question 7: I have stress caused by financial problems.	3.41 (1.20)	3.28 (1.23)	2.93 (1.18)

Note. SD = Standard Deviation; MMSE = Mini-Mental State Examination.

**Table 3:** Correlation matrix between Mini-Mental State Examination (MMSE) and life satisfaction with the financial attitude questions.

Variables	MMSE	Satisfaction with Life
1. MMSE	-	−0.02
2. Satisfaction with life	−0.02	-
3. Question 1: I am ready to face my financial future.	0.13	0.18 **
4. Question 2: I have enough financial security to face unforeseen expenses immediately.	−0.06	0.22 **
5. Question 3: I need outside help to manage my basic finances.	−0.02	−0.03
6. Question 4: I attend financial planning seminars/courses.	0.04	0.03
7. Question 5: I am able to rationally organize my budget for monthly expenses.	0.19 **	0.17 **
8. Question 6: I am able to pay my basic consumption.	0.22 **	0.27 **
9. Question 7: I have stress caused by financial problems.	−0.08	−0.10

Note. ** *p* < 0.01; MMSE = Mini-Mental State Examination.

## Data Availability

The datasets used and analyzed during the current study are available from the corresponding author upon reasonable request.
